# Effect of electroacupuncture at homotopic and heterotopic acupoints on abdominal pain in patients with irritable bowel syndrome: study protocol for a randomized controlled trial

**DOI:** 10.1186/s13063-018-2948-1

**Published:** 2018-10-16

**Authors:** Yanhui Peng, Hui You, Xiaoman Chen, Yanbing Chen, Yiling Yang, Jianpeng Huang, Nenggui Xu, Jianhua Liu

**Affiliations:** 1 The Secondary Medical College, Guangzhou University of Traditional Chinese Medicine, 111 Dade Road, Guangzhou, 510120 People’s Republic of China; 2 Guangzhou University of Traditional Chinese Medicine, 12 Jichang Road, Guangzhou, 510006 People’s Republic of China

**Keywords:** Electroacupuncture, IBS, DNIC, Homotopic acupoints, Heterotopic acupoints

## Abstract

**Background:**

Acupuncture has been widely applied in the clinic to treat irritable bowel syndrome (IBS), but the underlying mechanism remains unknown. Diffuse noxious inhibitory control (DNIC) is deficient in patients with IBS, which attenuates the systemic analgesic effect elicited by noxious stimulation that is remote from pain areas. Therefore, the aim of this study is to investigate the analgesic effect of electroacupuncture (EA) at homotopic or heterotopic acupoints on abdominal pain in patients with IBS.

**Methods/design:**

This study is a randomized, single-blinded, controlled, four-arm parallel trial. A total of 144 patients will be randomly assigned to four groups: a homotopic noxious stimulation group (group A), a homotopic innocuous stimulation group (group B), a heterotopic noxious stimulation group (group C), and a heterotopic innocuous stimulation group (group D). Each patient will receive 14 sessions of treatment, twice per week for 7 weeks. The primary outcome will be pain intensity measured with the visual analog scale. The secondary outcomes will include the IBS Symptom Severity Scale, IBS Quality of Life questionnaire, pain threshold (PT), and the Symptom Checklist-90 for psychological distress. The PT will be measured before and after every treatment. All other outcomes will be evaluated before the 1st treatment, after 7th and 14th treatment, and 3 months later during follow-up.

**Discussion:**

The aim of this study is to assess the analgesic effect of EA at homotopic (abdomen) acupoints and heterotopic (lower limb) acupoints on abdominal pain in patients with IBS, as well as the difference in analgesic effects between noxious and innocuous stimulation.

**Trial registration:**

Chinese Clinical Trial Registry, ChiCTR-IPR-15006879. Registered on 5 August 2015.

**Electronic supplementary material:**

The online version of this article (10.1186/s13063-018-2948-1) contains supplementary material, which is available to authorized users.

## Background

Irritable bowel syndrome (IBS) is a common functional gastrointestinal disease characterized by recurring symptoms of abdominal pain or discomfort, bloating, and altered bowel function in the absence of structural, inflammatory, or biochemical abnormalities [1]. It is reported that the prevalence of IBS varies from 7 to 21% worldwide [2]. The prevalence of IBS is higher in females than in males and is higher in patients younger than 50 years [3]. Among affected patients, abdominal pain is the most bothersome symptom and has the largest impact on quality of life [4, 5]. Since the pathogenesis of abdominal pain is still incompletely understood, the treatment is a challenging clinical problem [6, 7]. Many patients turn to complementary and alternative medicines, such as acupuncture [8].

Acupuncture has been clinically applied to treat acute and chronic pain for more than two millennia. Electroacupuncture (EA) is a modern adaptation of traditional Chinese acupuncture therapy. By adding direct current to the needles, the stimulation of acupoints can be amplified, which improves the therapeutic effect. EA is being increasingly used for pain relief for IBS. One clinical trial showed that EA is effective in lowering the severity and frequency of abdominal pain and increasing quality of life, compared with sham acupuncture [9]. Another previous trial suggested that EA may have positive effects on modulating pain sensations by altering the activation of neural pathways [10]. Animal experiments showed that EA attenuated visceral hyperalgesia through down-regulation of central serotonergic activities in the brain-gut axis [11]. The acupoint is the basis of acupuncture. The specific function of an acupoint is determined by the anatomical relationship between the disease focus and the segmental location of the acupoint [12]. Generally, acupoints may be classified as heterotopic or homotopic points, in terms of spinal segmental innervation patterns [13]. The pain-modulating effect of heterotopic and homotopic points involves different neuronal mechanisms. Innocuous stimulation applied to the pain area mainly excites large fibers (Aβ-fibers), exerting segmental inhibition through the spinal cord [14]. In contrast, noxious stimulation applied outside of the pain area activates small-diameter fibers (Aδ- and/or C-fibers), which produce extrasegmental analgesia through diffuse noxious inhibitory control (DNIC) [15].

Under normal circumstances, pain can be reduced by a conditioning noxious stimulus anywhere in the body; this is the definition of DNIC [16]. DNIC efficiency indicates the function of the endogenous pain modulation system for an individual patient [17]. DNIC function is critical for chronic pain prediction and treatment in clinical practice. However, deficient DNIC mechanisms have been found in various chronic pain conditions, including IBS [18], fibromyalgia [19], and temporomandibular joint disorder [18]. Impaired DNIC in patients with IBS was detected by King et al. by sensitivity to prolonged heat pain and the efficacy of pain inhibition. Compared with healthy controls, patients with IBS show increased pain sensitivity and reduced endogenous pain inhibition [18]. The selection of acupoints is an important consideration for clinical effects. Commonly, a combination of homotopic and heterotopic points has been used in IBS trials [9–11]. However, the difference between the effects of homotopic and heterotopic point stimulation is unknown.

Therefore, the aim of this study is to assess the analgesic effect of EA stimulation at homotopic (abdomen) and heterotopic (lower limbs) acupoints on abdominal pain in patients with IBS. Furthermore, we aim to determine which type of current intensity (noxious or innocuous) is the optimal stimulation for IBS clinical applications.

## Methods/design

### Study design

**Fig. 1:**
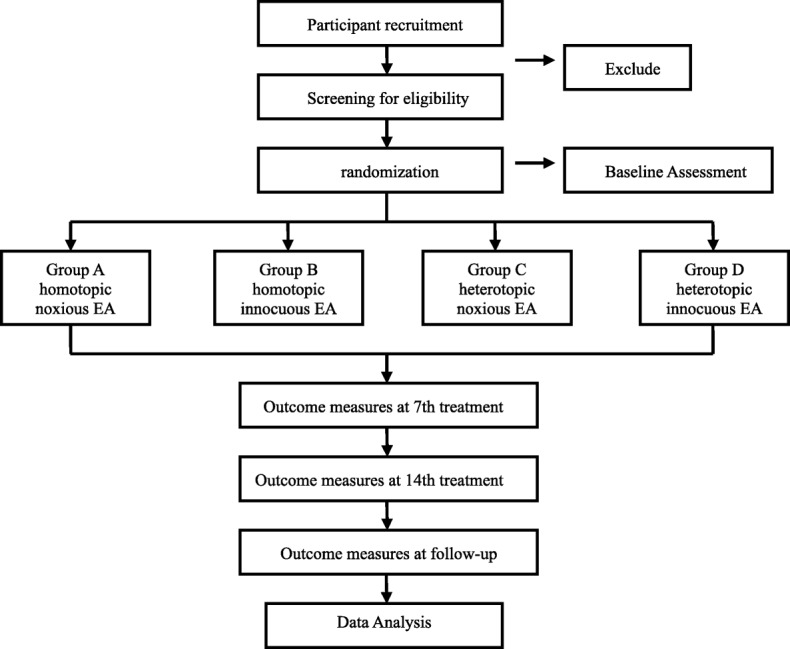
Flowchart of the study design. We will randomize 144 participants to the four groups. The interventions will last 30 min and will be carried out twice a week for 7 weeks. The study period will consist of the baseline, 7 weeks of treatment, and 3 months follow-up

**Fig. 2:**
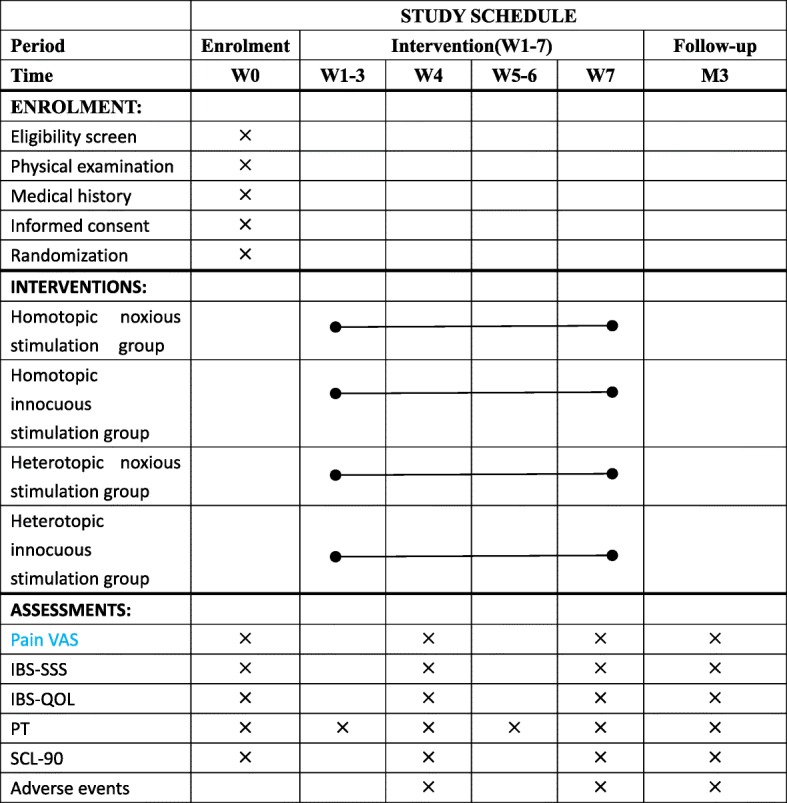
Study schedule (SPIRIT figure). The schedule of enrollment, allocation, interventions, and assessments. *W4*: the 7th treatment during the 4th week, *W7*: the 14th treatment during the 7th week

This study is a single-blind randomized controlled trial (RCT) that aims to compare the analgesic effect of two homotopic acupoint groups (noxious intensity and innocuous intensity) and two heterotopic acupoint groups (noxious intensity and innocuous intensity) for IBS. A total of 144 female patients will be recruited from the Guangdong Provincial Hospital of Traditional Chinese Medicine. Patients will be randomized to the homotopic noxious stimulation group (group A), the homotopic innocuous stimulation group (group B), the heterotopic noxious stimulation group (group C), or the heterotopic innocuous stimulation group (group D) in a 1:1:1:1 ratio. Patients will receive 14 sessions of EA treatment twice a week for 7 weeks. Each session will last 30 min. The flowchart of the trial is shown in Fig. 1. The study schedule is detailed in Fig. 2. The Standard Protocol Items: Recommendations for Interventional Trials (SPIRIT) checklist is provided as Additional file 1.

### Participants

#### Inclusion criteria

Patients who meet the following criteria are eligible for inclusion in the study: (1) they are females aged between 18 and 50 years, (2) they meet the criteria for IBS according to the Rome III diagnostic criteria, (3) IBS has been their main complaint for at least 6 months, (4) their average abdominal pain score has been greater than 30 mm (on a scale of 0–100 mm, with 0 indicating no pain and 100 the worst imaginable pain) in the past 2 weeks, and (5) they have signed an informed consent form.

#### Exclusion criteria

Patients will be excluded if they: (1) have a history of previous abdominal surgery related to IBS symptoms (other than appendectomy), (2) are currently taking antidepressant agents including tricyclic antidepressants and selective serotonin reuptake inhibitors, (3) have a history of usage of anticholinergic, antipsychotic, or antispasmodic drugs during the last 2 weeks, (4) are currently participating in another trial or have enrolled in a trial during the past month, (5) are currently pregnant or planning to become pregnant, (6) have a Hamilton Depression Rating Scale (HAMD) count of 21 points or more, (7) have a serious concomitant disease of the heart, liver, or kidney, or have diabetes, (8) have a blood system disease such as thrombocytopenia with bleeding tendency.

#### Recruitment

All participants will be recruited through advertisements on notice boards in the Traditional Chinese Medicine Hospital of Guangdong Province. If a patient is interested in participating she will contact the researcher by WeChat or telephone. Then the patient will be screened based on the inclusion and exclusion criteria in a face-to-face interview with the researcher. The researcher is expected to explain the study in detail, including the potential benefits and risks. Once a participant agrees to join the RCT, she will be asked to sign an informed consent form before randomization.

### Randomization

We plan to use the random block method. A designated researcher who has no contact with any participant will conduct the randomization. The random number table is generated by the “completely random design” program of the Package for the Encyclopedia of Medical Statistics for Windows (PEMS 3.1, West China School of Public Health, Sichuan, China). All participants will be assigned to four groups with a 1:1:1:1 allocation ratio. The random sequence will be generated in a block size of 4. The researcher then will make randomization assignment cards and put the cards into sequentially numbered, opaque envelopes. According to the time order in which participants pass the screening test, another researcher will arrange the patients into different groups and inform the acupuncturists of the group assignment.

### Blinding

In this RCT procedure, the patients, data collection staff, and data analysts will be blinded. However, the acupuncturist knows the treatment allocation due to the nature of the acupuncture manipulation. During the intervention, the acupuncturist and the data collection researcher will be separated immediately after the treatment starts and are instructed not to exchange information. Data collection staff and data analysts have no information about grouping or treatment.

### Interventions

All EA treatments will be performed by one acupuncturist who has more than 10 years of experience and a Chinese medicine practitioner license. The treatments will be performed according to the standard protocol already prepared. All participants will take a supine position on the treatment couch to receive treatment. Each participant will receive 14 sessions of treatments, twice a week for 7 weeks. Each session will last for 30 min. No additional treatment is allowed.

All selected patients will be randomized to four groups: group A will receive EA at homotopic acupoints with noxious current intensity, which will be 30% above the patient’s pain threshold. Group B will receive EA at homotopic acupoints with innocuous current intensity, which will be 30% below the patient’s pain threshold. Group C will receive EA at heterotopic acupoints with noxious current intensity. Group D will receive EA at heterotopic acupoints with innocuous current intensity. Thus, group A is called the homotopic noxious group, group B is called the homotopic innocuous group, group C is called the heterotopic noxious group, and group D is called the heterotopic innocuous group. We selected the homotopic acupoints ST25 (Tianshu) and ST26 (Wailing), and the heterotopic acupoints ST36 (Zusanli) and ST37 (Shangjuxu), because these acupoints are most commonly used to treat IBS in China [20].

After local area disinfection, sterile, disposable needles (length 50 mm, diameter 0.30 mm; Huatuo, Suzhou Medical Supply Factory Co., Ltd., Suzhou, China) will be inserted to a depth of 25–40 mm. The acupuncturist will manipulate the needle to achieve a “De-qi” sensation for the patient. ST25 will be connected to the same-side ST26 in group A and group B. ST36 will be connected to the same-side ST37 in group C and group D. The reason behind the connection is to form a circuit containing a HANS-200E stimulator (Nanjing Jisheng Co., China) for 30 min. The frequency in all four groups will be the same as an alternating wave with a frequency of 2/100 Hz. The treatment details for the four groups are presented in Table 1.

**Table 1:** Details of treatment in the four groups

Group	Points	Stimulation parameters
Group A	Tianshu (ST25) Wailing (ST26)	Current intensity is 30% above the patient’s pain threshold. Alternating wave with a frequency of 2/100 Hz
Group B	Tianshu (ST25) Wailing (ST26)	Current intensity is 30% below the patient’s pain threshold. Alternating wave with a frequency of 2/100 Hz
Group C	Zusanli (ST36) Shangjuxu (ST37)	Current intensity is 30% above the patient’s pain threshold. Alternating wave with a frequency of 2/100 Hz
Group D	Zusanli (ST36) Shangjuxu (ST37)	Current intensity is 30% below the patient’s pain threshold. Alternating wave with a frequency of 2/100 Hz

### Outcome assessments

#### Primary outcome measurement

##### 2.6.1.1. Abdominal pain intensity (0–100 mm visual analog scale (VAS))

Each patient will rate her abdominal pain from the previous week on a 0–100 mm scale, with 0 indicating no pain and 100 the worst imaginable pain. According to a previous study [21], the reliability and validity of the VAS in patients with IBS has been established.

##### 2.6.1.2. IBS Symptom Severity Scale (IBS-SSS)

This questionnaire consists of five questions summing to a score of 500 points. The five questions concern abdominal pain intensity, abdominal pain frequency, abdominal distension degree, defecation satisfaction, and interference with quality of life. SSS intensity is assessed by the total score. If the score is lower than 75, the patient is considered to be in remission. When the score is 75–175, 175–300, and above 300, we will consider the patient to be mild, moderate, and severe, respectively. The reliability and validity of the IBS-SSS for treatment has been verified in many studies [22, 23].

#### Secondary outcomes measurement

##### 2.6.2.1. IBS Quality of Life (IBS-QOL) questionnaire

The IBS-QOL questionnaire is composed of eight dimensions, with 34 items assessing the degree to which IBS interferes with the patient’s quality of life. The eight-dimension self-assessment includes dysphoria (eight items), interference with activity (seven items), body image (four items), health concerns (three items), food avoidance (eight items), social reaction (eight items), sex (eight items), and relationships (three items). Each item is evaluated on a 5-point Likert scale (score 1–5). The total score on the IBS-QOL ranges from 34 to 170. Higher scores indicate worse quality of life. The scale has achieved high validity and reliability with IBS patients in previous studies [24].

##### 2.6.2.2. Pain threshold (PT)

PT is a secondary outcome in this study, and is a more objective measure of pain than the VAS. The PT will be tested by a digital algometer (EP601C, East China Normal University of Science and Technology, Guangxi, China) immediately before and after each treatment. The most obviously tender point (Ashi point) on the abdomen will be tested. The patient will lie in a supine position on the treatment couch. The algometer has a 1-cm^2^ rubber probe, which will be pressed vertically against the abdominal wall. The trained assessor will position the algometer perpendicular to the tender point and apply gradual pressure at a steady rate of approximately 0.5 kg/cm^2^/s. The patient will request the treatment to be stopped when the applied pressure becomes painful, and the value on the digital algometer will be recorded. The pressure pain threshold will be measured three times at each point, with the average value collected for analysis.

##### 2.6.2.3. The Symptom Checklist-90 (SCL-90)

Psychological status will be assessed by the SCL-90 [25], which comprises 10 symptom factors: somatization, obsessive-compulsive disorder, interpersonal sensitivity, depression, anxiety, hostility, phobic anxiety, paranoid ideation, psychoticism, and other. The sum of the 10 component scores will be the final score. Higher scores indicate poorer mental status.

### Follow-up

All participants will be asked to return in 3 months for follow-up. The researcher will contact participants via telephone to collect therapeutic effect information after the treatment has been completed for 3 months.

#### 2.8. Sample size

The trial aims to detect a difference in analgesic effect between the four parallel groups. There are insufficient previous data regarding VAS scores for the treatment of IBS using EA. Therefore, we could not calculate the appropriate sample size according to the sample size calculation formula. We plan to enroll 144 patients, with 36 individuals in each group, allowing for 20% attrition. The outcomes of this pilot study will aid in the calculation of the appropriate sample size for further randomized clinical trials.

#### 2.9. Adverse events

All patients will be asked to report any adverse events during the trial. Possible adverse events include local pain, bleeding, local infection, palpitations, fainting, and other symptoms. The researcher will address and record all adverse events. Once a serious adverse event occurs, the researcher may need to terminate the trial and submit a written report to the Research Ethics Committee immediately.

### 2.10. Quality control

To guarantee the quality of this study, we will run several simulations with our colleagues playing the role of patients to identify any problems with our study protocol before the study begins. The one researcher who tests the PT will be trained many times to improve consistency of results. To guarantee the objectivity of the data, effect assessment and statistics will be blinded during the study period. Finally, all details will be recorded on a patient case report form (CRF).

#### 2.11. Data management and monitoring

All patient data will be recorded on the CRF at each visit. Two blinded data collection staff will collect the CRFs and perform double-data entry. A qualified clinical trial expert will monitor the entire process of this RCT. The Clinical Research Center of Guangdong Hospital of Traditional Chinese Medicine will check the CRFs periodically to ensure the quality and timeliness of data.

### 2.11. Statistical analyses

The statistical analyses will be performed by a statistician who will be blinded to the treatments and study protocol. All data will be analyzed according to intention-to-treat (ITT) principles to reduce deviation. Non-continuous variables will be analyzed by chi-square test. All normally distributed continuous variables will be described by mean ± standard error (SEM). The paired *t* test will be used to compare the outcome measurements before and after treatment. The differences between group and time will be analyzed by repeated measures analysis of variance (ANOVA). If we find a significant overall difference among the four groups for a particular outcome, we will use the post hoc test (Newman-Keuls test) to adjust for multiple comparisons to determine the statistical differences among pairwise groups. All analyses will be conducted with SPSS (version 21.0) software. A *P* value of 0.05 or less will be considered statistically significant.

## Discussion

At present, the most well-known clinical application of acupuncture in the world is for chronic pain. However, many specific questions about the therapeutic effect of acupuncture also exist. It is generally believed that multifaceted factors influence its curative effect, among which acupoint selection is vital. It is well known that each point has its particular therapeutic indication, and acupuncture at different acupoints shows different effects. Therefore, more trials are needed to investigate differences in acupoint selection. Most studies on acupoint selection have focused on traditional Chinese medicine (TCM) theory or the treatment effects of acupuncture. However, few studies have used DNIC to select the appropriate points and optimal EA intensity for clinical practice.

DNIC in patients with IBS has been shown to be decreased compared to healthy controls. A high-quality study showed that patients with IBS failed to show pain suppression during a DNIC session and showed little decrease in pain [18]. In a similar finding, heterotopic acupoint stimulation cannot induce significant acupuncture analgesia. At this point, EA may not be effective. Therefore, we hypothesize that homotopic acupoints (ST25 and ST26) will have better effects than heterotopic acupoints (ST36 and ST37). The real result will help guide us in choosing effective acupoints (homotopic or heterotopic acupoints) to treat abdominal pain.

Furthermore, we will try to screen the optional current intensity for EA clinical application. In this study, the effect of noxious EA and innocuous EA will be compared in two homotopic groups and two heterotopic groups. Innocuous intensity EA stimuli have been demonstrated to only produce local analgesia by segmental inhibition. They cannot produce heterotopic pain-relieving effects by activating the DNIC. In contrast, noxious intensity EA stimuli not only can produce local analgesia but can also excite small afferent fibers through DNIC mechanisms to produce heterotopic analgesia [12]. We expect that homotopic groups will show better effects than heterotopic groups, but we do not know whether segmental noxious EA will be more effective than segmental innocuous EA. A series of clinical trials have studied EA current intensity [26, 27]; however, there has been no clear conclusion to date. The suitable intensity of EA may vary according to the patient’s physiological status and disease [28]. Based on previous studies, we set the current intensity to 30% above and below each patient’s pain threshold to indicate noxious and innocuous stimulation, respectively [29].This study will help identify a suitable EA intensity for IBS.

Studies have demonstrated that EA at different frequencies can release different kinds of neuropeptides. A combination of 2 Hz and 100 Hz (2/100 Hz) stimulation produces all four opioid peptides simultaneously, which results in a maximal analgesic effect. This finding has been verified clinically in patients with chronic pain, including patients with low back pain and diabetic neuropathy [30]. We thus chose 2/100 Hz as the best frequency for this study.

In conclusion, this trial will provide significant evidence of the principles (homotopic or heterotopic) for selecting acupoints for EA analgesia in chronic pain conditions with impaired DNIC. In addition, it will help us identify a suitable current intensity for EA stimulation.

### Trial status

This trial is currently recruiting patients. It will be completed by 15 May 2019.

## Additional file

Additional file 1: SPIRIT checklist. (DOC 117 kb)

## Abbreviations

ANOVA: Analysis of variance
CRF: Case report form
DNIC: Diffuse noxious inhibitory control
EA: Electroacupuncture
IBS: Irritable bowel syndrome
IBS-QOL: IBS Quality of Life (questionnaire)
IBS-SSS: IBS Symptom Severity Scale
ITT: Intention-to-treat
PT: Pain threshold
SCL-90: Symptom Checklist-90 for psychological distress
TCM: Traditional Chinese medicine
